# Experimental exchange of grins between quantum Cheshire cats

**DOI:** 10.1038/s41467-020-16761-0

**Published:** 2020-06-15

**Authors:** Zheng-Hao Liu, Wei-Wei Pan, Xiao-Ye Xu, Mu Yang, Jie Zhou, Ze-Yu Luo, Kai Sun, Jing-Ling Chen, Jin-Shi Xu, Chuan-Feng Li, Guang-Can Guo

**Affiliations:** 1 CAS Key Laboratory of Quantum Information, University of Science and Technology of China, Hefei, 230026 China; 2 CAS Centre For Excellence in Quantum Information and Quantum Physics, University of Science and Technology of China, Hefei, 230026 China; 3 Theoretical Physics Division, Chern Institute of Mathematics, Nankai University, Tianjin, 300071 China

**Keywords:** Quantum optics, Quantum mechanics, Single photons and quantum effects

## Abstract

Intuition suggests that an object should carry all of its physical properties. However, a quantum object may not act in such a manner—it can temporarily leave some of its physical properties where it never appears. This phenomenon is known as the quantum Cheshire cat effect. It has been proposed that a quantum object can even permanently discard a physical property and obtain a new one it did not initially have. Here, we observe this effect experimentally by casting non-unitary imaginary-time evolution on a photonic cluster state to extract weak values, which reveals the counterintuitive phenomenon that two photons exchange their spins without classically meeting each other. A phenomenon presenting only in the quantum realm, our results are in stark contrast with the perception of inseparability between objects and properties, and shed new light on comprehension of the ontology of observables.

## Abstract (web summary)

The quantum Cheshire cat effect has already highlighted how, in the quantum realm, a physical property can be temporarily detached from an object. Here, the authors go a step further, experimentally demonstrating how a particle's spin can be permanently separated and exchanged with that of another particle.

## Introduction

Quantum paradoxes^1^, which exhibit counterintuitive phenomena, have provided multiple perspectives in study of the fundamentals of quantum mechanics, from Bell non-locality^2^, quantum contextuality^3^ to macro-realism^4^. Recently, the quantum Cheshire cat Gedankenexperiment, presented by Aharonov et al.^5^, illustrated the counterintuitive phenomenon that a physical property can be disembodied from its physical carrier, akin to the scene A grin without a cat in the story Alice in Wonderland^6^. In the context of quantum Cheshire cat, the possibility of isolating an object from its physical properties^7^ has sparked the interest of theoretical physicists. Soon after, extensions and further discussions of more general scenarios were presented^8–12^. Also, the effect of quantum Cheshire cat has been found to have intrinsic links to other quantum paradoxes: for instance, the three-box paradox^13^.

In principle, experimental observation of the quantum Cheshire cat effect can be implemented in following perspectives: first, because the physical property is isolated from its carrier, changes in that property should not affect the evolution of the carrier, provided that the final quantum interference is not disturbed. Second, if one have the ability to measure the “cat” and “grin” observables, the effect can also be deduced from the measured values. With the help of significant advances in weak measurement techniques achieved in various quantum systems, experimental observation of the quantum Cheshire cat effect has been presented in neutronic^14^ and photonic system^15^. However, while these observations were based on true quantum objects (i.e., single neutrons or photons), the results do not strictly support the unique quantum character of the cat, because completely identical phenomena can be observed with classical light^16^. Although it is commonly accepted that quantum interference plays a crucial role in the quantum Cheshire cat effect^17^, experiments involving only the first-order interference cannot resolve the question of whether, in the sense of quantum mechanics, the grin of the “Cheshire cat” is left behind.

In this article, we report an experiment with manifoldly entangled photons that demonstrates a stronger object–property separation, whereby an object can permanently drop a certain property and acquire a property that it did not have from another object^11^. The photons are taken as “Cheshire cats”, and their polarisations as “grins”. A bilayer Franson interferometer is exploited to post-select over the desired ensemble. The trails of the cats and grins can be exposed by weak values for the corresponding observables, from which it is deduced that both of the grins are first separated from their carrier cats and then swapped. The required weak values are extracted by means of perturbation, more explicitly, adding various kinds of density filters to cast imaginary-time evolution (ITE) on the system, which spares the need of introducing an additional pointer to a complex system for weak value extraction. This method may also find its usage in other fields such as contextuality-based quantum computation^18,19^ and quantum metrology^20^. The results overcome the main criticism of previous experiments that the separation can also be observed in classical systems, and that the disembodiment is temporary. The apparent separation of the physical properties from the quantum objects, and the exchanges of these properties lucidly exhibit the genuine quantum feature of the quantum Cheshire cats.

## Results

### Schematic illustration

**Fig. 1:**
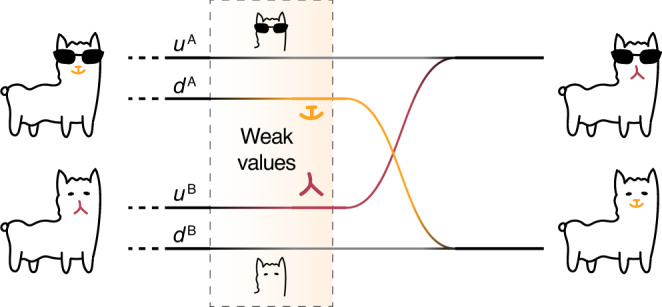
**Schematic illustration**. A non-contacting grin exchange takes place between two quantum Cheshire cats. The cats Anna with sunglasses and Belle without sunglasses independently enter two bilayer channels, where the sundered grins from the cats can be revealed by their weak values. The structure of the channels is twisted so the two paths for Anna and Belle denoted by *u* and the two denoted by *d* ultimately join each other, respectively. Consequently, after exiting the setup, both cats are astonished to find that they have swapped grins without having met each other.

The narrative of two Cheshire cats exchanging their grins is plotted in Fig. 1. The creatures of Cheshire cats can freely take off their grin in Wonderland, but are not allowed to do so in the real world. Two Cheshire cats named Anna and Belle are spawned at distant locations. Each of them enters the Wonderland via a one-way channel denoted by the grey line and sends its grin forward via another one-way channel denoted by the coloured line. The separation can be revealed by a courier called “weak value”. The cats do not expect that the mischievous designer leads Anna’s coloured grin to appear on Belle and vice versa. Upon returning to reality, Anna gets Belle’s grin and has to put it on to avoid being faceless. This weird story may actually take place in the quantum realm. A photon, which is a spin-1 boson, is forbidden from being observed without spin. However, it can be separated from its spin during a quantum process, as is well demonstrated in quantum Cheshire cat experiments^14–16^. By manipulating the channels of photons and their spins to twist the internal link, we can deterministically swap the spins of two photons while preventing them from appearing at the same site.

### Theoretical layout

A set of properly defined observables can help describe the perplexing behaviors of quantum Cheshire cats. The path (“cat”) observables, revealing the spatial positions of the cats, read ${\Pi }_{{u}}\equiv \left|{u}\right\rangle \left\langle {u}\right|$ and ${\Pi }_{{d}}\equiv \left|{d}\right\rangle \left\langle {d}\right|$, with *u* and *d* denoting the two possible paths a Cheshire cat can take. Both of the projectors have an eigenvalue of one to indicate the existence of the cat, and an eigenvalue of zero to indicate the absence of the cat in the corresponding path. The spin observable is given by the Pauli operator ${\sigma }_{z}=\left|\uparrow \right\rangle \left\langle \uparrow \right|-\left|\downarrow \right\rangle \left\langle \downarrow \right|$, which has a pair of eigenvalues  ±1, with ↑ and ↓ denoting the smile and frown of a Cheshire cat. To detect the exact spin in one path (“grin”, e.g., in the upper path), a product of the two observables should be introduced, which reads *σ*_*z*_ ⊗ *Π*_*u*_. The product observable has three eigenvalues:  ±1 and 0, corresponding to the eigenstates $\left|\uparrow \right\rangle \left|{u}\right\rangle$, $\left|\downarrow \right\rangle \left|{u}\right\rangle$ and the degenerate subspace spanned by $\left|\uparrow \right\rangle \left|{d}\right\rangle$ and $\left|\downarrow \right\rangle \left|{d}\right\rangle$, respectively.

The quantum Cheshire cat effect is best witnessed via the weak values of the cat and grin observables. The weak value of an observable *O* with respect to pre-selection $\left|\xi \right\rangle$ and post-selection $\left\langle \zeta \right|$ is defined as^21^:

1 $${\left\langle O\right\rangle }_{{w}}=\frac{\left\langle \zeta | O| \xi \right\rangle }{\left\langle \zeta | \xi \right\rangle },$$

which describes the conditioned average of the observable for a pre-/post-selected ensemble. By associating the locations of observables to their corresponding weak values, tracing physical object and properties in a specific process has been made possible^14,22^. Precisely, for an observable with spectrum of 0 and  ±1, a null weak value ${\left\langle O\right\rangle }_{{w}}=0$ indicates that the property represented by the observable is not in presence; a unit weak value ${\left\langle O\right\rangle }_{{w}}=1$, conversely, indicates that the corresponding property is here. The weak values may have anomalous behaviors. For instance, the settings in the first proposed quantum Cheshire cat Gedankenexperiment^5^ leads to ${\left\langle {\Pi }_{u}\right\rangle }_{{w}}={\left\langle {\sigma }_{z}\otimes {\Pi }_{d}\right\rangle }_{{w}}=1$ and ${\left\langle {\Pi }_{d}\right\rangle }_{{w}}={\left\langle {\sigma }_{z}\otimes {\Pi }_{u}\right\rangle }_{{w}}=0$, so a cat and its grin is discovered at different sites, creating an apparent object–property separation.

What happens to the cats in Fig. 1 successfully passing through the pre- and post-selection? For this ensemble, Anna has travelled through the path marked by *d,* whereas Belle has taken the path *u*; the spin of Anna appears in the path *u*, whereas the spin of Belle appears in the path *d*. It follows the above properties of weak value that:

2 $${\left\langle {\Pi }_{\mu }^{\nu }\right\rangle }_{{w}}={\delta }_{\mu {d}}{\delta }_{\nu {A}}+{\delta }_{\mu {u}}{\delta }_{\nu {B}},$$

3 $${\left\langle {\sigma }_{z}^{\nu }\otimes {\Pi }_{\mu }^{\nu }\right\rangle }_{{w}}={\delta }_{\mu {u}}{\delta }_{\nu {A}}+{\delta }_{\mu {d}}{\delta }_{\nu {B}},$$

here, the *δ* symbol is the Kronecker-*δ* function, *μ* ∈ {*u*, *d*} denotes possible paths and *ν* ∈ {*A*, *B*} indexes the cat names. Given the set of weak values, when the two channels marked by *u* combine, the input modes, namely, the spin from Anna and the body from Belle, have to join each other to fire a detector. A similar event takes place on the channels marked by *d*, where Anna acquires Belle’s spin. In other words, each of the two quantum Cheshire cats deterministically swaps grin with its counterparts.

One may ask whether the particular configuration described above can even be arranged. Interestingly, entanglement in quantum theory supplies the required catalyst here, that the two cats can indeed exchange their grins even they can never be found at the same location. To observe such a counterintuitive phenomenon, the system should be pre-selected as a four-qubit linear cluster state $\left|\xi \right\rangle =[-\left|{\Phi }^{-}\right\rangle \otimes \left|{{u}}^{{A}}{{d}}^{{B}}\right\rangle +\left|{\Phi }^{+}\right\rangle \otimes \left|{{d}}^{{A}}{{u}}^{{B}}\right\rangle ]/\sqrt{2}$, and the ensembles for post-selection are accordingly chosen to be $\left|\zeta \right\rangle ={\left|D\right\rangle }^{\otimes 2}\otimes \left|{\Psi }^{-}\right\rangle$, with $\left|D\right\rangle =(\left|\uparrow \right\rangle +\left|\downarrow \right\rangle )/\sqrt{2}$. Here, the states before and after the direct product symbol correspond to the system’s “cat” and “grin” degrees of freedom. $\left|{\Phi }^{\pm }\right\rangle$ and $\left|{\Psi }^{\pm }\right\rangle$ represent the celebrated Bell states, explicitly defined as ${\left|{\Phi }^{\pm }\right\rangle }_{{\rm{grin}}}=\frac{1}{\sqrt{2}}(\left|{\uparrow }^{{A}}{\uparrow }^{{B}}\right\rangle \pm \left|{\downarrow }^{{A}}{\downarrow }^{{B}}\right\rangle )$, and ${\left|{\Psi }^{\pm }\right\rangle }_{{\rm{cat}}}=\frac{1}{\sqrt{2}}(\left|{{u}}^{{A}}{{\rm{d}}}^{{\rm{B}}}\right\rangle \pm \left|{{d}}^{{A}}{{u}}^{{B}}\right\rangle )$. The superscripts “*A*” and “*B*” are indices for cats Anna and Belle. The exchange of grins can be verified by substituting $\left|\xi \right\rangle$ and $\left\langle \zeta \right|$ into (1) to immediately recover (2) and (3). Observing that ${\Pi }_{{u}}^{{A}}{\Pi }_{{u}}^{{B}}\left|\xi \right\rangle ={\Pi }_{{d}}^{{A}}{\Pi }_{{d}}^{{B}}\left|\xi \right\rangle =0$, such behavior is strictly forbidden for a localised classical object because it invokes action at a distance, so the Cheshire cats here are purely from the quantum realm.

### Weak value extraction

It is argued that the quantum Cheshire cat effect is nothing other than an optical illusion. As a strong measurement corresponding to an operator projects the system onto its eigenspace by Lüder’s rule, a series of non-commuting sequential measurements that do not share all eigenvectors cannot reveal meaningful information about the original state. However, a disturbance-friendly measurement (i.e., weak measurement), can actually reveal the paradox, which can be witnessed from the weak values of the observables extracted from the pre- and post-selected ensembles^5^.

**Fig. 2:**
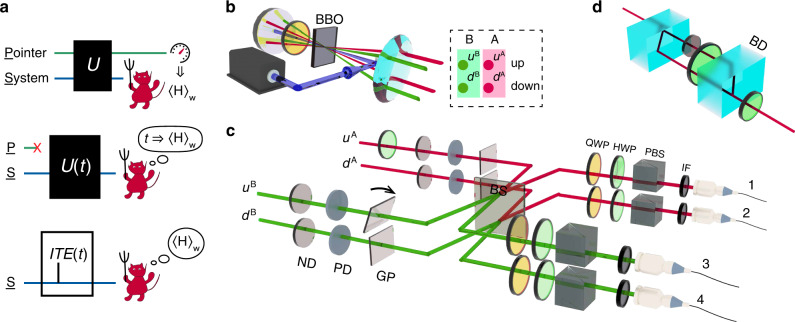
**Design of experiments**. **a** Concept of weak value extraction. Top: rigorous weak measurement. The system and pointer are entangled by coupling evolution ${\mathcal{U}}=\exp (-i{\mathcal{H}}t)$ with the interaction Hamiltonian being ${\mathcal{H}}={O}_{{\rm{s}}}\otimes P$. The demon cast judicious choices of post-selection to obtain the weak value ${\left\langle {O}_{{\rm{s}}}\right\rangle }_{{w}}$. Mid: interpretation of weak value without pointer. The system's evolution ${{\mathcal{U}}}_{s}$ due to a perturbation is characterised by weak value in the weak interaction regime^23^. Bottom: weak value extraction based on perturbation. A linear relationship between the post-selection probability and the interaction time of ITE can be established, whose incline gives the real part of the weak value ${\left\langle O\right\rangle }_{{w}}$. **b** Photon source. Biphoton (central wavelength 813.4 nm), four-qubit hyperentangled state is generated via type-I spontaneous parametric down-conversion (SPDC) process by pumping a *β*-barium borate (BBO) crystal twice in a confocal structure^24,25^. CM concave mirror, QWP quarter-wave plate. **c** The main setup to exchange grins. Neutral density filter (ND) and polarisation-sensitive density filter (PD) implement the perturbation on the path observable *Π* and the conditional spin observable *σ*_*z*_ ⊗ *Π*, respectively. A polarisation independent BS, followed by four polarisation analysers, each composed of a set of quarter- or half-wave plates (QWP, HWP) and a PBS, implements post-selection. Four 0.2-mm-thick glass plates (GPs) are inserted in the four arms with one of them rotatable to compensate the biphoton phase. The photons are filtered by 3-nm-bandwidth interference filters (IF) with a central wavelength of 813.4 nm before collected by four single-mode fibres and guided to single-photon detectors. **d** A PD is constructed from two polarisation beam displacers (BDs) and an ND filter inserted in one arm.

The quantum measurement procedure, including von Neumann measurement, positive operator-valued measurement and weak measurements, requires an auxiliary pointer object for readout^26,27^. See Fig. 2a, to extract the weak value ${\left\langle {O}_{s}\right\rangle }_{{w}}$, the standard procedure using weak measurement weakly couples the system to a pointer with a Hamiltonian $\tilde{{\mathcal{H}}}={O}_{s}\otimes P$, where the subscript *s* indicates that *O*_*s*_ only acts on the system, and *P* is the pointer’s momentum operator. One then strongly collapses the system to some judiciously chosen target states. Because the pointer is now entangled with the system by interaction $\tilde{{\mathcal{U}}}=\exp (-i\tilde{{\mathcal{H}}}t)$, it will be steered to different conditional output states, enabling the calculation of corresponding weak value^21^.

Are there alternative approaches to extract the weak value other than weak measurements? Recent advances have given affirmative answers. For instance, in the work of directly measuring an entangled biphoton state^28^, the authors read out the required weak values of non-local observables via strong measurements and the concept of modular value^29^. Besides, a linear relationship exists between the probability of successful post-selection and the strength of a unitary perturbation. Consequently, the imaginary part of the weak value can be linked to the linear model’s slope^23^. In this approach, no ancillary pointer is needed and the interaction of weak measurement can be effectively substituted by some perturbations in a weak manner where quantum coherence can still be preserved.

For an observation of quantum Cheshire cats, schemes of weak value extraction with the feature of resource-saving are quite preferred, as both the photons’ polarisation and path degrees of freedom have been consumed. Because the coefficients in the pre- and post-selection rays in the scheme are real, the weak values for both the “cat” and the “grin” observables have no imaginary part. Following the insight of ref. ^23^, here we establish a linear relationship between the probability of successful post-selection and the strength of perturbations with the form of ITE, and exploit this relationship to characterise the weak values without introducing any auxiliary pointer states. The non-unitary ITE evolution generated by an observable *O* reads ${\mathcal{U}}({\mathcal{H}},t)={e}^{-{\mathcal{H}}t}$, where ${\mathcal{H}}=O$ is the Hamiltonian of the system and the interaction time *t* → 0 is an analogue to the coupling strength in the system–pointer coupling process. Explicitly, let ${N}_{0}=| \left\langle \xi | \zeta \right\rangle {| }^{2}$ and $N({\mathcal{U}})=| \left\langle \zeta | {\mathcal{U}}| \xi \right\rangle {| }^{2}$ be the probabilities of conducting a successful post-selection before and after applying the perturbation, then, the real part of the weak value can be linked to the probability correction, that:

4 $${\left.\frac{\partial }{\partial t}\frac{N({\mathcal{U}})}{{N}_{0}}\right|}_{t\to 0}=-2{\rm{Re}}{\left\langle O\right\rangle }_{{w}}.$$

The detailed proof is presented in the “Methods” section. Consequently, when ITEs with sufficiently small interaction times (see Supplementary Note 2) are imposed on the pre-selection state, a linear relationship exists between the probability correction and the interaction time, and the model’s slope is proportional to the real part of the weak value of the ITE’s generating Hamiltonian.

### Experimental implementation

We will demonstrate the exchange of grins between quantum Cheshire cats with an optical setup. Two entangled photons are adopted as the quantum Cheshire cats. Utilising the photon’s intrinsic pseudo-spin degree of freedom, namely, the polarisation, the smile ($\left|\uparrow \right\rangle$) and frown ($\left|\downarrow \right\rangle$) of the cats are represented by photon’s horizontal ($\left|H\right\rangle$) and vertical ($\left|V\right\rangle$) polarisation. To prepare the desired ensembles, a hyperentanglement biphoton source and a bilayer Franson interferometer are exploited. Each photon has two possible paths, one in the upper layer of the Franson interferometer and one in the lower layer. They correspond to the path states $\left|u\right\rangle$ and $\left|d\right\rangle$, respectively.

The photon source of the experimental setup is shown in Fig. 2b. A vertically polarised ultraviolet laser (*λ* = 406.7 nm) was focused on a type-I cut *β*-barium borate (BBO) crystal. The degenerate spontaneous parametric down-conversion (SPDC) process allows horizontally polarised photon pair emission at *λ* =  813.4 nm along two opposite rays on a conical surface. Both the laser and the down-converted photons’ wavefunction pass through a QWP (*λ*/4 at 813.4 nm, optical axis oriented at 45°), reflect off a spherical (*f* = 150 mm) mirror, pass through the wave plate and are focused again on the BBO crystal. The polarisation of down-converted photons is rotated to vertical by double-passing the QWP, and the SPDC process can again produce horizontally polarised photon pairs. Finally, a positive lens (*f* = 150 mm) transforms the conical parametric emission to cylindrical.

Due to the system’s confocal structure and small dispersion, the wavefunctions of down-converted photons from successive pumping processes are spatially and temporally overlapped, thereby producing an entanglement ring. Selecting two pairs of opposite points on the ring results in biphoton polarisation-path hyperentangled states^25^. The photon on the left side is chosen as “Anna” and that on the right side is called “Belle”. The hyperentangled state reads ${\left|{\Phi }^{+}\right\rangle }_{{\rm{pol}}}\otimes {\left|{\Psi }^{+}\right\rangle }_{{\rm{path}}}$, which is further converted to the cluster state $\left|\xi \right\rangle$ by inserting a half-wave plate (HWP) with its optical axis oriented at 0° into Anna side’s upper interferometer arm, as shown in Fig. 2c. The post-selection on $\left|\zeta \right\rangle$ is conducted by first superimposing Anna and Belle’s wavefunction on the upper and lower layers, respectively, on a beam splitter (BS), and then picking out the diagonal polarisation with a set of quarter-wave plates (QWPs) and HWPs, followed by a polarising beam splitter (PBS). The spatial wavefunction is maximally entangled for the Franson interference occurring on the BS (more details are given in the Supplementary Note 5).

To implement the perturbation of ITE and acquire the weak values, a neutral density (ND) filters or polarising dependent (PD, only attenuates vertically polarised components) filter is inserted into one of the four arms of the interferometer before recombining the photons on the BS, and its position is slightly adjusted to block different portions of light, effectively changing *t*_*n*(*p*)_. The corresponding Hamiltonians are ${\Pi }_{\mu }^{\nu }$ and ${({\mathbb{1}}-{\sigma }_{z})}^{\nu }/2\otimes {\Pi }_{\mu }^{\nu }$, respectively. So, an ND filter serves to obtain the path weak value ${\langle {\Pi }_{\mu }^{\nu }\rangle }_{{w}}$, and the spin weak value ${\langle {\sigma }_{z}^{\nu }\otimes {\Pi }_{\mu }^{\nu }\rangle }_{{w}}$ is linked to the system’s behaviors under both kinds of filter insertion. Moreover, let us define the intensity transmissivity *γ*_*n*_, *γ*_*p*_ of the filter as the intensity ratio of an unpolarised light after and before passing through the neutral and polarising filter, respectively. Then, the interaction time *t*_*n*(*p*)_ can be associated by *γ*_*n*(*p*)_ by ${\gamma }_{{n}}={e}^{-2{t}_{{n}}}$ and ${\gamma }_{{p}}=(1+{e}^{-2{t}_{{p}}})/2$. Note that the definition for polarising filter is equivalent with a 2*γ*_*p*_ − 1 and unity transmissivity for vertically and horizontally polarised photons, respectively. Also, let ${N}_{\mu ,{n}({p})}^{\nu }$ denote the ratio of coincidence rates after and before neutral or polarising filter insertion in the path *μ* of photon *ν*. Substituting the definitions of Hamiltonians to (4) yields:

5 $${\left\langle {\Pi }_{\mu }^{\nu }\right\rangle }_{{w}}=-\frac{1}{2}\frac{\partial {N}_{\mu ,{n}}^{\nu }}{\partial {t}_{{n}}},$$

6 $${\left\langle {\sigma }_{z}^{\nu }\otimes {\Pi }_{\mu }^{\nu }\right\rangle }_{{w}}=-\frac{1}{2}\frac{\partial {N}_{\mu ,{n}}^{\nu }}{\partial {t}_{{n}}}+\frac{\partial {N}_{\mu ,{p}}^{\nu }}{\partial {t}_{{p}}}.$$

The detailed proof of (6) goes to the “Methods” section.

**Fig. 3:**
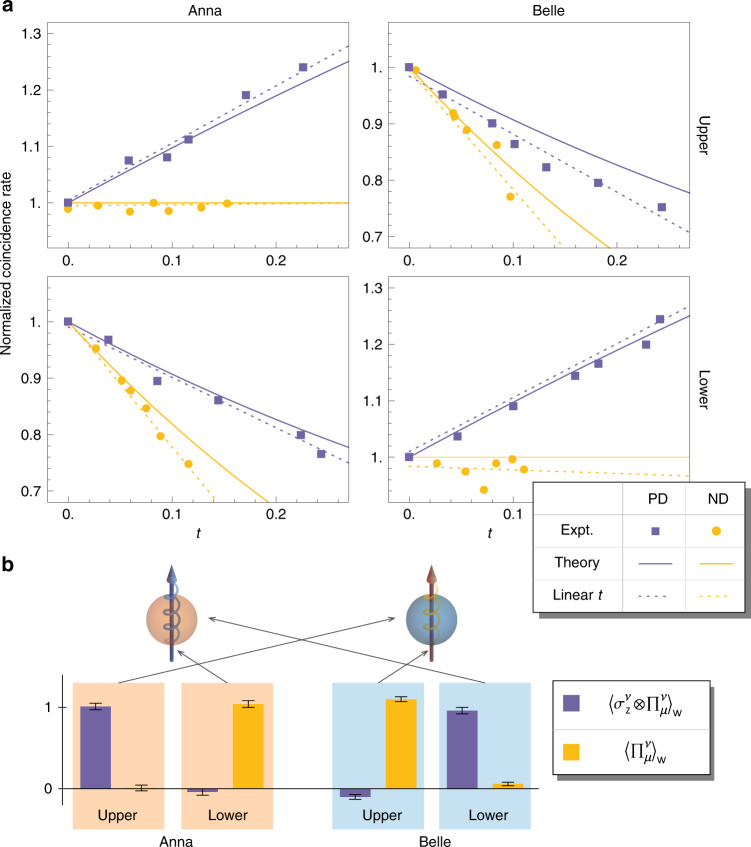
**Experimental results**. **a** Recorded data with linear fitted model and theoretical curve of the counting rates normalised with respect to when no filters are inserted, plotted against the interaction time of the attenuators in four arms of the interferometer. The data and curve with violet colour represent the case of ND filter insertion, and marigold colour for the case of PD filter. The error bars, calculated assuming Poissonian counting statistics, are smaller than data points and thus not shown. **b** Weak values calculated from the slope of the linear fitted models and an illustration of the photons swapping their spins. Given the calculated weak values for each port, the implication is that Annaʼs photon is in the lower port but the spin is measured in the upper port, and vice versa for Belle. Consequently, on the beam splitter, Anna receives Belleʼs grin when the two lower paths converge. Similarly, Belle captures Annaʼs grin on the intersection of two upper paths.

Finally, the eight weak values ${\langle {\Pi }_{\mu }^{\nu }\rangle }_{{w}}$ and ${\langle {\sigma }_{z}^{\nu }\otimes {\Pi }_{\mu }^{\nu }\rangle }_{{w}}$ are deduced by filtering the photons in one arm by different ratios, recording the corresponding counting rates, and least square fitting the linear model (6) with recorded data points of coincidence against transmissivity, whose slope can be related to the weak values. The brightness of the cluster state is about 1 × 10^3^ counts per second, and integration time for each data point is 100 s. The rate of dark coincidence events is of the magnitude of 10^−2^ Hz. The precise implementation of pre- and post-selection (see Supplementary Notes 3 and 5) guarantees the weak values have negligible imaginary parts. For each data point, the count of coincident events ${N}_{\mu ,{n}({p})}^{\nu }$ is normalised by the value when the filter is not inserted, and the *N*-*t* data points are asymptotically fitted to a linear model in the neighbourhood of *t*_*n*(*p*)_ → 0, so the required derivatives are given by the model’s slope. The eight groups of recorded ${N}_{\mu ,{n}({p})}^{\nu }$-*t* points and their linear fitted models are plotted in Fig. 3. From the models’ slopes, the weak values are deduced to be ${\left\langle {\Pi }_{{u}}^{{A}}\right\rangle }_{{w}}=-0.01(3)$, ${\left\langle {\Pi }_{{d}}^{{A}}\right\rangle }_{{w}}=1.04(4)$, ${\left\langle {\Pi }_{{u}}^{{B}}\right\rangle }_{{w}}=1.11(4)$, ${\left\langle {\Pi }_{{d}}^{{B}}\right\rangle }_{{w}}=0.06(4)$, ${\left\langle {\sigma }_{{u}}^{{A}}\right\rangle }_{{w}}=1.01(3)$, ${\left\langle {\sigma }_{{d}}^{{A}}\right\rangle }_{{w}}=-0.04(4)$, ${\left\langle {\sigma }_{{u}}^{{B}}\right\rangle }_{{w}}=0.10(2)$, and ${\left\langle {\sigma }_{{d}}^{{B}}\right\rangle }_{{w}}=0.04(3)$. The counting events for photon detection follow Poisson distribution, and following this statistic, the standard deviation for weak values given in the parentheses is numerically estimated via Monte Carlo simulation. The calculated values are consistent with the theoretical values, and shows that for the successfully post-selected ensemble, Anna always comes from the lower layer, whereas its spin effectively comes from the upper layer. The case of Belle is contrary, that the photon is from the upper layer with its spin separated on the lower layer. Finally, on the BS, Anna eventually receives the spin of Belle, and Belle captures Anna’s spin.

## Discussion

The usefulness and genuine quantum property of quantum Cheshire cat have been intensely debated. Introducing second-order interference may provide some insight into these topics. Comparing with the original quantum Cheshire cat proposal with only first-order interference, the observed effects in this demonstration are significantly more robust, because entanglement provides additional resilience against local disturbances. For example, when a local disturbance of the bit flip error is applied on one of the arms of the interferometer, the phenomenon of quantum Cheshire cat is not affected, that the spins can still not be detected on the interferometer arms where the photons can be detected (proof details are given in Supplementary Note 4). This result is not valid for first-order demonstrations, where the Cheshire cat is more prone to disturbance^17^. Moreover, when the bit flip error is applied to the arms with ${\langle {\sigma }_{z}^{\nu }\otimes {\Pi }_{\mu }^{\nu }\rangle }_{{w}}=0$, the weak values observed in (2) and (3), together with the final counting rate, are left unchanged. This observation supports the proposal^5^, that the unwanted disturbance can be completely removed in a post-selected manner by producing quantum Cheshire cats, where the affected observables are confined out of the regions with disturbance. Hopefully, its application may also be found in the tasks about quantum communication, for example, teleporting a spin through a noisy channel.

Regarding the genuine quantum property of quantum Cheshire cats, although quantum objects are used in previous demonstrations, the observed effects did not exclude the interpretation with other languages such as classical electrodynamics^16^. Conversely, the entangled photons, together with the Franson interference exploited in this demonstration, manifest Bell-type non-locality and reject any classical description. By unveiling the nature of quantum Cheshire cat, the stereotype that a property must faithfully belong to an object is overthrown and becomes another counterintuitive, seemingly paradoxical phenomenon provided by the quantum theory.

We have observed the phenomenon of grin swapping between two quantum Cheshire cats, which only appears in a pre- and post-selected entangled quantum system. The required weak values are acquired with high accuracy by casting ITE in a linear optics setup. Our experiment should help foster new research in the area of quantum information and inspire new ideas regarding the ontology of physical properties beyond the dependent object.

## Methods

### Proof of the perturbation method

In this section, we derive the link between the probability of successful post-selection and the interaction time in the context of ITE. The result for a unitary perturbation was already described in ref. ^23^. Originating from Wick rotation in special relativity^30^, the ITE operation is applied in, e.g., quantum field theory^31^ and quantum simulations^32^. The parameter *t* is not generally restricted, however, in this scheme it has to be sufficiently small to resemble a vanishing interaction time and guarantee minimal disturbance of the system.

Recall that for ITE the relation between non-unitary operation and the Hamiltonian is ${\mathcal{U}}({\mathcal{H}},t)=\exp (-{\mathcal{H}}t)$. Assuming weak interaction *t* → 0, the detection probability of a pre- and post-selected event perturbed by ITE (with Maclaurin series up to the first order of *t*) reads:

7 $$\begin{array}{c}N({\mathcal{U}})=| \left\langle \zeta | {\mathcal{U}}| \xi \right\rangle {| }^{2}=| \left\langle \zeta | (1-Ot+...)| \xi \right\rangle {| }^{2}\\ \quad \, \, ={N}_{0}-2t{\rm{Re}}\left\langle \xi | \zeta \right\rangle \left\langle \zeta | O| \xi \right\rangle +{\mathcal{O}}({t}^{2}),\end{array}$$

where in the last term the big-${\mathcal{O}}$ notation is adopted, which is not to be confused with the operator *O* that generates the ITE. By dividing both sides of Eq. (7) by *N*_0_ and taking partial derivative with respect to *t*:

8 $${\left.\frac{\partial }{\partial t}\frac{N({\mathcal{U}})}{{N}_{0}}\right|}_{t\to 0}=-2{\rm{Re}}\left(\frac{\left\langle \zeta | O| \xi \right\rangle }{\left\langle \zeta | \xi \right\rangle }\right)=-2{\rm{Re}}{\left\langle O\right\rangle }_{{w}}.$$

From Eq. (8), the real part of the weak value ${\left\langle O\right\rangle }_{{w}}$ is half of the additive inverse of the derivative of the detection probability normalised by its undisturbed value, with respect to the interaction time *t*. In this demonstration, all parameters in pre- and post-selection states and the ITE non-unitary evolution operator are real numbers, so all weak values have to be purely real. From now on we do not distinguish the weak value from its real part.

For location measurement at path *μ* of photon *ν*, the Hamiltonian is taken to be ${{\mathcal{H}}}_{\mu ,{n}}^{\nu }={\Pi }_{\mu }^{\nu }$, the corresponding ITE operator acting on the state vector is ${{\mathcal{U}}}_{\mu ,{n}}^{\nu }({{\mathcal{H}}}_{\mu ,{n}}^{\nu },{t}_{{n}})={\mathbb{1}}-{\Pi }_{\mu }^{\nu }(1-{e}^{-{t}_{{n}}})$. The operation complying with this ITE is to decrease the photon number in photon *ν*’s path *μ*. Explicitly, an ND filter with transmissivity ${\gamma }_{{n}}={e}^{-2{t}_{{n}}}$ is introduced to partially block this path, which multiplies the coincidence rates by ${N}_{\mu ,{n}}^{\nu }$. It follows from (8) that (omitting *t* → 0):

9 $${\left\langle {\Pi }_{\mu }^{\nu }\right\rangle }_{{w}}=-\frac{1}{2}\frac{\partial {N}_{\mu ,{n}}^{\nu }}{\partial {t}_{{n}}}.$$

Similarly, for spin measurement at path *μ* of photon *ν* the Hamiltonian is chosen to be ${{\mathcal{H}}}_{\mu ,{p}}^{\nu }={({\mathbb{1}}-{\sigma }_{z})}^{\nu }\otimes {\Pi }_{\mu }^{\nu }/2$. Then, ${{\mathcal{U}}}_{\mu ,{p}}^{\nu }({{\mathcal{H}}}_{\mu ,{p}}^{\nu },t)={\mathbb{1}}-{\Pi }_{\mu }^{\nu }{({\mathbb{1}}-{\sigma }_{z})}^{\nu }(1-{e}^{-{t}_{{p}}})/2$, experimentally, this corresponds to a PD filter insertion, multiplying the coincidence rates by ${N}_{\mu ,{p}}^{\nu }$. By substituting the form of ITE operator into (8) and taking advantage of (9):

10 $${\left\langle {\Pi }_{{d}}^{\nu }\right\rangle }_{{w}}-{\left\langle {\sigma }_{z}^{\nu }\otimes {\Pi }_{\mu }^{\nu }\right\rangle }_{{w}}=-\frac{\partial {N}_{\mu ,{p}}^{\nu }}{\partial {t}_{{p}}},$$

11 $${\left\langle {\sigma }_{z}^{\nu }\otimes {\Pi }_{\mu }^{\nu }\right\rangle }_{{w}}=-\frac{1}{2}\frac{\partial {N}_{\mu ,{n}}^{\nu }}{\partial {t}_{{n}}}+\frac{\partial {N}_{\mu ,{p}}^{\nu }}{\partial {t}_{{p}}}.$$

Observing that the transmissivity for vertically and horizontally polarised photons are ${e}^{-2{t}_{{p}}}$ and 1 respectively, ${\gamma }_{{p}}=(1+{e}^{-2{t}_{{p}}})/2$ again correlates the interaction time with the experimentally measurable transmissivity.

### Polarising density filter

For the measurement of photon spin information, the required PD filter is a synthetic element. The formation of the PD filter is shown in Fig. 2d. A pair of BDs, together with two HWPs separate and reconverges the horizontally and vertically polarised wavefunction. An ND filter on the vertically polarised path filters a fraction of photons to mimic the change of *t*_*p*_.

## Supplementary information

Supplementary Information

## Data Availability

The data that support the findings of this study are available from the authors upon request.
