# Supplementary material for: Experimental exchange of grins between quantum Cheshire cats
Source: Nat Commun. 2020 Jun 15;11:3006. doi: 10.1038/s41467-020-16761-0 (PMC7295756; doi:10.1038/s41467-020-16761-0)
Supplement: Supplementary file 1 — Supplementary Information [file 41467_2020_16761_MOESM1_ESM.pdf]

Supplementary Information - Experimental Exchange of Grins between Quantum Cheshire Cats

Zheng-Hao Liu, Wei-Wei Pan, Xiao-Ye Xu, Mu Yang, Jie Zhou, Ze-Yu Luo,
Kai Sun, Jing-Ling Chen, Jin-Shi Xu, Chuan-Feng Li, and Guang-Can Guo

SUPPLEMENTARY NOTE 1. RELATION BETWEEN WEAK VALUE, FILTER TRANSMISSIVITY AND COUNTING RATE.

The interaction time is related to density filter's transmissivity by $\gamma_n = e^{-2t_{\rm n}}$ and $\gamma_p = (1 + e^{-2t_{\rm p}})/2$. The relation of normalised counting rate and interaction time has been provided in the main text. Here we show that the former can also be directly related to the transmissivity by applying the chain rule for derivatives:

$$\begin{aligned}\langle \Pi^{\nu}_{\mu} \rangle_{\rm w} &= -\frac{1}{2}\frac{\partial N^{\nu}_{\mu,{\rm n}}}{\partial t_{\rm n}} = -\frac{1}{2}\frac{d\gamma_{\rm n}}{dt_{\rm n}}\frac{\partial N^{\nu}_{\mu,{\rm n}}}{\partial \gamma_{\rm n}} \\ &= \gamma_{\rm n}\frac{\partial N^{\nu}_{\mu,{\rm n}}}{\partial \gamma_{\rm n}} \approx \frac{\partial N^{\nu}_{\mu,{\rm n}}}{\partial \gamma_{\rm n}}. \end{aligned} \tag{1}$$

Where the approximation at the last step holds because the weak interaction limit $t \to 0$ yields $\gamma \to 1$. For the spin measurement,

$$\begin{aligned}\langle \Pi^{\nu}_{\mu} \rangle_{\rm w} - \langle \sigma^{\nu}_{z} \otimes \Pi^{\nu}_{\mu} \rangle_{\rm w} &= -\frac{\partial N^{\nu}_{\mu,{\rm p}}}{\partial t_{\rm p}} \\ &= -\frac{d\gamma_{\rm p}}{dt_{\rm p}}\frac{\partial N^{\nu}_{\mu,{\rm p}}}{\partial \gamma_{\rm p}} = (2\gamma_{\rm p} - 1)\frac{\partial N^{\nu}_{\mu,{\rm p}}}{\partial \gamma_{\rm p}}, \end{aligned} \tag{2}$$

$$\begin{aligned}\langle \sigma^{\nu}_{z} \otimes \Pi^{\nu}_{\mu} \rangle_{\rm w} &= (1 - 2\gamma_{\rm p})\frac{\partial N^{\nu}_{\mu,{\rm p}}}{\partial \gamma_{\rm p}} + \gamma_{\rm n}\frac{\partial N^{\nu}_{\mu,{\rm n}}}{\partial \gamma_{\rm n}} \\ &\approx -\frac{\partial N^{\nu}_{\mu,{\rm p}}}{\partial \gamma_{\rm p}} + \frac{\partial N^{\nu}_{\mu,{\rm n}}}{\partial \gamma_{\rm n}}. \end{aligned} \tag{3}$$

Supplementary Equations (1) and (3) establish the relation between postselection probability and transmissivity of filters, with the interaction time t serves as intermediate variable.

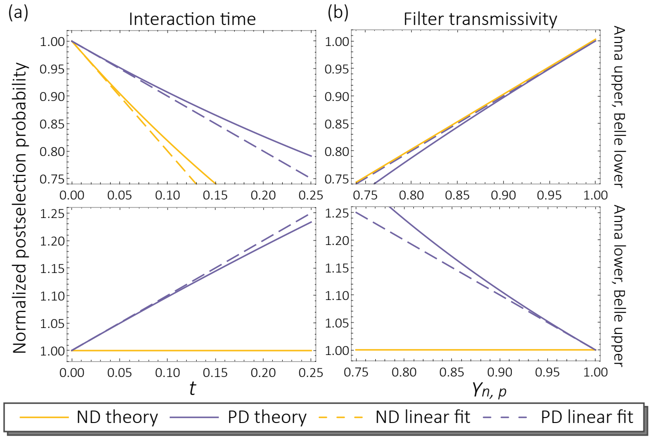

Supplementary Figure 1. Comparison of theoretical curve and approximation methods. (a) calculated coincidence rate as a function of interacting time parameter t, plotted along with their tangent at $t = 0$. (b) calculated coincidence rate as a function of filter tranmissivity, $\gamma_{\rm n(p)}$, plotted along with their tangent at $\gamma = 1$.

SUPPLEMENTARY NOTE 2. ACCURACY OF THE APPROXIMATION METHOD.

The validity of the two proposed approximation method is checked by comparing the theoretical curve and tangent model in the limit of vanishing interaction time and unity transmissivity. The results are plotted in Supplementary Figure 1. It can be seen that as the interaction time increases, the fitted lines describing the weak values start to differ from theoretical predictions. The transmissivity method introduce no systematic error for extracting weak values of path observables. Notwithstanding, in the experiment, we stick to the first approach for less steps of approximation used and overall faithful fitting.

The effective interaction time in our experiment falls in the region $t_{\rm n} \leq 0.13$ and $t_{\rm p} \leq 0.26$, which implies that the interaction time is sufficiently short so the slope of the linear fitted model resembles the derivative of the theoretical curve with acceptable accuracy.

SUPPLEMENTARY NOTE 3. CHARACTERISATION OF THE PHOTON SOURCE.

The tomographic result from maximum likelihood estimation is presented in Supplementary Figure 2 for the biphoton states retrieved from Anna's lower arm and Belle's upper arm, as well as the states retrieved from Anna's upper arm and Belle's lower arm, which is modi-

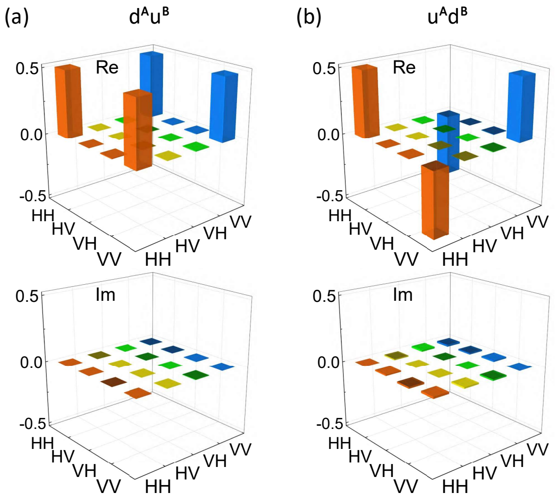

Supplementary Figure 2. Characterising the photon source with the reconstructed biphoton density matrices (DMs) from state tomography without the BS in the postselection system. (a-b) The tomographic result at detectors 2-4, which represents the polarisation state on $|{\rm d^A u^B}\rangle_{path}$, and at 1-3 representing polarisation for $|{\rm u^A d^B}\rangle_{path}$. An almost perfect $|\Phi^{+}\rangle$ state for the former and $|\Phi^{-}\rangle$ for the latter, the calculated DMs have fidelities of 0.990 and 0.992 with target DMs respectively.

fied by an HWP set at 0°. The two photons' polarisation properties is characterised by using the polarisation discrimination system just before the detectors and perform state tomography. In this step, the BS is temporarily removed, allowing the coincidence between detectors 1 and 4 to detect the polarisation state in $|\mathrm{u}\rangle^{\mathrm{A}} |\mathrm{d}\rangle^{\mathrm{B}}$ path, and 2 and 3 to reveal $|\mathrm{d}\rangle^{\mathrm{A}} |\mathrm{u}\rangle^{\mathrm{B}}$. See Supplementary Figure 2, These results resemble maximally entangled polarisation state, with the former exhibiting 99.0% fidelity with $|\Phi^+\rangle$ and the latter 99.2% with $|\Phi^-\rangle$.

SUPPLEMENTARY NOTE 4. RESILIENCE OF TWO-PHOTON QUANTUM CHESHIRE CATS UNDER LOCAL FILP ERROR.

Here we prove the proposition in Discussion section of the main text. A bit flip error acting on path μ of photon ν can be expressed as $\mathcal{E}^{\nu}_{\mu}(t) = (t\sigma^{\nu}_{x} + (1-t)\mathbb{1}) \otimes \Pi^{\nu}_{\mu} + \mathbb{1} \otimes (\mathbb{1} - \Pi^{\nu}_{\mu})$. where t is a continuous parameter representing the strength of disturbation ranging in $[0, 1]$.

As $|\xi\rangle = [-|\Phi^-\rangle \otimes |\mathrm{u}^{\mathrm{A}}\mathrm{d}^{\mathrm{B}}\rangle + |\Phi^+\rangle \otimes |\mathrm{d}^{\mathrm{A}}\mathrm{u}^{\mathrm{B}}\rangle]/\sqrt{2}$, the perturbed state after the bit flip channel reads:

$$\mathcal{E}^{\mathrm{A}}_{\mathrm{u}}(t)\,|\xi\rangle = [(-(1-t)\,|\Phi^-\rangle + t\,|\Psi^-\rangle) \otimes |\mathrm{u}^{\mathrm{A}}\mathrm{d}^{\mathrm{B}}\rangle + |\Phi^+\rangle \otimes |\mathrm{d}^{\mathrm{A}}\mathrm{u}^{\mathrm{B}}\rangle]/\sqrt{2}, \tag{4}$$

$$\mathcal{E}^{\mathrm{A}}_{\mathrm{d}}(t)\,|\xi\rangle = [-|\Phi^-\rangle \otimes |\mathrm{u}^{\mathrm{A}}\mathrm{d}^{\mathrm{B}}\rangle + ((1-t)\,|\Phi^+\rangle + t\,|\Psi^+\rangle) \otimes |\mathrm{d}^{\mathrm{A}}\mathrm{u}^{\mathrm{B}}\rangle]/\sqrt{2}, \tag{5}$$

$$\mathcal{E}^{\mathrm{B}}_{\mathrm{u}}(t)\,|\xi\rangle = [-|\Phi^-\rangle \otimes |\mathrm{u}^{\mathrm{A}}\mathrm{d}^{\mathrm{B}}\rangle + ((1-t)\,|\Phi^+\rangle + t\,|\Psi^+\rangle) \otimes |\mathrm{d}^{\mathrm{A}}\mathrm{u}^{\mathrm{B}}\rangle]/\sqrt{2}, \tag{6}$$

$$\mathcal{E}^{\mathrm{B}}_{\mathrm{d}}(t)\,|\xi\rangle = [(-(1-t)\,|\Phi^-\rangle - t\,|\Psi^-\rangle) \otimes |\mathrm{u}^{\mathrm{A}}\mathrm{d}^{\mathrm{B}}\rangle + |\Phi^+\rangle \otimes |\mathrm{d}^{\mathrm{A}}\mathrm{u}^{\mathrm{B}}\rangle]/\sqrt{2}. \tag{7}$$

Substituting (7) and $\langle\zeta| = \langle D|^{\otimes 2} \otimes (\mathrm{u}^{\mathrm{A}}\mathrm{d}^{\mathrm{B}} - \mathrm{d}^{\mathrm{A}}\mathrm{u}^{\mathrm{B}})/\sqrt{2}$ into the definition of weak values yields:

	$\langle\Pi^{\mathrm{A}}_{\mathrm{u}}\rangle_{\mathrm{w}}$	$\langle\Pi^{\mathrm{A}}_{\mathrm{d}}\rangle_{\mathrm{w}}$	$\langle\Pi^{\mathrm{B}}_{\mathrm{u}}\rangle_{\mathrm{w}}$	$\langle\Pi^{\mathrm{B}}_{\mathrm{d}}\rangle_{\mathrm{w}}$	$\langle\sigma^{\mathrm{A}}_{z} \otimes \Pi^{\mathrm{A}}_{\mathrm{u}}\rangle_{\mathrm{w}}$	$\langle\sigma^{\mathrm{A}}_{z} \otimes \Pi^{\mathrm{A}}_{\mathrm{d}}\rangle_{\mathrm{w}}$	$\langle\sigma^{\mathrm{B}}_{z} \otimes \Pi^{\mathrm{B}}_{\mathrm{u}}\rangle_{\mathrm{w}}$	$\langle\sigma^{\mathrm{B}}_{z} \otimes \Pi^{\mathrm{B}}_{\mathrm{d}}\rangle_{\mathrm{w}}$
Applying $\mathcal{E}^{\mathrm{A}}_{\mathrm{u}}(t)$	0	1	1	0	$1-2t$	0	0	1
Applying $\mathcal{E}^{\mathrm{A}}_{\mathrm{d}}(t)$	0	1	1	0	1	0	0	1
Applying $\mathcal{E}^{\mathrm{B}}_{\mathrm{u}}(t)$	0	1	1	0	1	0	0	1
Applying $\mathcal{E}^{\mathrm{B}}_{\mathrm{d}}(t)$	0	1	1	0	1	0	0	$1-2t$

Supplementary Table I. The weak values of observables after applying perturbation.

So by directly checking these numerical values, one finds that regardless of the exact location where the perturbation is imposed, an interferometer arm cannot have simultaneous nonzero cat and grin weak values, which proved the robustness of quantum Cheshire cats against local bit flip error. The similar result cannot be obtained in the original Cheshire cats proposal, where the filp error will always result in simultaneous nonzero cat and grin weak values in one of the arms.

Furthermore, the results after applying $\mathcal{E}^{\mathrm{A}}_{\mathrm{d}}$ and $\mathcal{E}^{\mathrm{B}}_{\mathrm{u}}$ are identical to the formula (2) and (3) in the main text, which means that the error is virtually shielded by the postselection process. This conclusion directly support the proposition by Aharonov et al. in the original Cheshire cat paper, where the authors suggest to remove unwanted disturbance by producing quantum Cheshire cats and postselecting over desired ensembles.

SUPPLEMENTARY NOTE 5. ANALYSING EFFECT OF BIPHOTON PHASE.

To appreciate the mechanism of the interferometer, one recall that all possible input states fall in the Hilbert space spanned by $|\psi^{\pm}\rangle_{p}$. On the BS,

$$|\Psi^+\rangle_{\mathrm{path}} \overset{BS}{\to} i\,(|\mathrm{u}\rangle^{\mathrm{A}} |\mathrm{d}\rangle^{\mathrm{A}} + |\mathrm{d}\rangle^{\mathrm{B}} |\mathrm{u}\rangle^{\mathrm{B}})/\sqrt{2}, \tag{8}$$

$$|\Psi^-\rangle_{\mathrm{path}} \overset{BS}{\to} -(|\mathrm{u}\rangle^{\mathrm{A}} |\mathrm{d}\rangle^{\mathrm{B}} - |\mathrm{d}\rangle^{\mathrm{A}} |\mathrm{u}\rangle^{\mathrm{B}})/\sqrt{2}, \tag{9}$$

so only the singlet spatial state is registered as coincident counting between Alice and Bob. However, experimentally the difference between biphoton phase have to be taken into account. To ensure the postselection on path qubits picks out the singlet state $|\Psi^-\rangle_{\mathrm{path}}$, the biphoton phase between $|\mathrm{u}^{\mathrm{A}}\mathrm{d}^{\mathrm{B}}\rangle$ and $|\mathrm{d}^{\mathrm{A}}\mathrm{u}^{\mathrm{B}}\rangle$ terms have to be aligned. The effect of biphoton phase can be stated a phase factor θ in spatial mode postselection $\langle\Psi(\theta)| = \langle\mathrm{d}^{\mathrm{A}}\mathrm{u}^{\mathrm{B}}| - e^{i\theta}\langle\mathrm{u}^{\mathrm{A}}\mathrm{d}^{\mathrm{B}}|$. Recall that the preselected state is

$$\begin{aligned}|\xi\rangle =& (-|H^{\mathrm{A}}H^{\mathrm{B}}\rangle + |V^{\mathrm{A}}V^{\mathrm{B}}\rangle) \otimes |\mathrm{u}^{\mathrm{A}}\mathrm{d}^{\mathrm{B}}\rangle/2 \\ &+ (|H^{\mathrm{A}}H^{\mathrm{B}}\rangle + |V^{\mathrm{A}}V^{\mathrm{B}}\rangle) \otimes |\mathrm{d}^{\mathrm{A}}\mathrm{u}^{\mathrm{B}}\rangle/2 \\ =& [-|\Phi^-\rangle \otimes |\mathrm{u}^{\mathrm{A}}\mathrm{d}^{\mathrm{B}}\rangle + |\Phi^+\rangle \otimes |\mathrm{d}^{\mathrm{A}}\mathrm{u}^{\mathrm{B}}\rangle]/\sqrt{2},\end{aligned} \tag{10}$$

and $\langle\mathrm{d}^{\mathrm{A}}\mathrm{d}^{\mathrm{B}}|\Phi^-\rangle = 0$, the $\langle D|^{\otimes 2}$ polarising projection neglects $|\Phi^-\rangle_{\mathrm{pol}}$ terms in $|\xi\rangle$, so virtually only the $|\mathrm{d}^{\mathrm{A}}\mathrm{u}^{\mathrm{B}}\rangle$

term arrives at post-selection, and scanning biphoton phase does not introduce interference fringes.

A ND filter with transmissivity γ_n shifts the preselected state to

$$|\xi^{\nu}_{\mu,n}\rangle = [-\sqrt{\gamma_{\rm n}}^{\delta_{\mu u}\delta_{\nu \rm A}+\delta_{\mu d}\delta_{\nu \rm B}}\,|\Phi^-\rangle\otimes|{\rm u^A d^B}\rangle \tag{11}$$

$$+\sqrt{\gamma_n}^{\delta_{\mu d}\delta_{\nu \rm A}+\delta_{\mu u}\delta_{\nu \rm B}}\,|\Phi^+\rangle\otimes|{\rm d^A u^B}\rangle]/\sqrt{2}. \tag{12}$$

Where $\mu \in \{u, d\}$ and $\nu \in \{\rm A, B\}$ indicates the location of filter. Because only $|{\rm d^A u^B}\rangle$ term contribute to postselection, the ND filter in Belle's upper arm or Anna's lower arm will cause a substantial decrease of total coincidence, while one locating in Anna's upper arm or Belle's lower arm has no effect. The prediction are experimentally verified and plotted in Supplementary Figure 3a.

When the filter is polarising-dependent, the following calculation shows that the biphoton phase may affect the probability of final postselection. The preselected state biased by the PD filter of transmissivity γ_p reads

$$|\xi^{\nu}_{\mu,\rm p}\rangle = \begin{cases} -(|H^{\rm A}H^{\rm B}\rangle - |V^{\rm A}V^{\rm B}\rangle)\otimes|{\rm u^A d^B}\rangle/2 + (|H^{\rm A}H^{\rm B}\rangle + \sqrt{2\gamma_p-1}\,|V^{\rm A}V^{\rm B}\rangle)\otimes|{\rm d^A u^B}\rangle/2, & \delta_{\mu d}\delta_{\nu \rm A}+\delta_{\mu u}\delta_{\nu \rm B}=1, \\ -(|H^{\rm A}H^{\rm B}\rangle - \sqrt{2\gamma_p-1}\,|V^{\rm A}V^{\rm B}\rangle)\otimes|{\rm u^A d^B}\rangle/2 + (|H^{\rm A}H^{\rm B}\rangle + |V^{\rm A}V^{\rm B}\rangle)\otimes|{\rm d^A u^B}\rangle/2, & \delta_{\mu u}\delta_{\nu \rm A}+\delta_{\mu d}\delta_{\nu \rm B}=1. \end{cases} \tag{13}$$

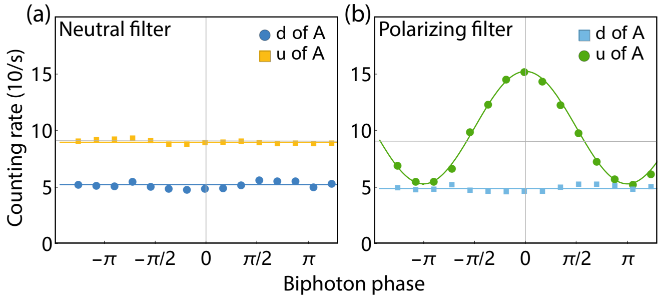

Supplementary Figure 3. Measurement result of the Franson interference. The interference curves are used to tune the postselection system in this work. The error bars, calculated assuming Poissonian counting statistics, are smaller than data point and is not shown. (a) Counting rate versus scanning biphoton phase, with a ND filter inserted in Anna's upper or lower path. Whereas total coincidence decreases in the latter case, no evident interference fringe is observed, because the post-selection for polarisation overshadows the interference term. (b) Counting rate versus scanning biphoton phase, with a polarisation-dependent density filter inserted in Anna's upper or lower path. Partially blocking the vertically-polarised component in Anna's upper path renders the interference term relevant, which is utilised to accurately align the biphoton phase.

For the first case, i.e, a PD filter in Anna's lower arm or Belle's upper arm, the spin singlet in $|{\rm u^A d^B}\rangle$ term is not disturbed, and it is still rejected by postselection $\langle D|^{\otimes 2}$. Consequently, no interference fringe is observed, and the total coincidence rate is reduced due to decreased wavefunction amplitude in $|{\rm d^A u^B}\rangle$ term. For the second case, i.e, a PD filter in Anna's upper arm or Belle's lower arm, the polarisation state of $|{\rm u^A d^B}\rangle$ term is biased from $|\Phi^-\rangle_{\rm pol}$, so interference appears between the two spatial modes. As shown in Fig. 3b, a clear sinusoidal curve can be observed for coincidence rate between detectors 2 and 3 when the GP is tilted to modulate the biphoton phase.

The biphoton phase between two pairs of possible paths is adjusted by inserting and tilting GPs before BS before conducting the measurements, and is finely tuned in order to ensure the postselection on path qubits picks out the antisymmetric state $|\Psi^-\rangle_{\rm path}$. By noticing that $|\langle D|^{\otimes 2}\langle\Phi(\theta)|\xi^{\rm A}_{\rm u,p}\rangle|^2$ is maxed out at $\theta=0$, this calibration can be accomplished by inserting a PD filter in the upper arm on Anna's side, and tilting one of the GPs to maximise the coincident rate. Fig. 3 plots the counting rate curve against biphoton phase which originates from this Franson interference.
